# Ectomycorrhizal-Dominated Boreal and Tropical Forests Have Distinct Fungal Communities, but Analogous Spatial Patterns across Soil Horizons

**DOI:** 10.1371/journal.pone.0068278

**Published:** 2013-07-09

**Authors:** Krista L. McGuire, Steven D. Allison, Noah Fierer, Kathleen K. Treseder

**Affiliations:** 1 Department of Biology, Barnard College, Columbia University, New York, New York, United States of America; 2 Department of Ecology & Evolutionary Biology, University of California Irvine, Irvine, California, United States of America; 3 Department of Earth System Science, University of California Irvine, Irvine, California, United States of America; 4 Department of Ecology & Evolutionary Biology, University of Colorado, Boulder, Colorado, United States of America; 5 Cooperative Institute for Research in Environmental Sciences, University of Colorado, Boulder, Colorado, United States of America; CNRS - Université Lyon 1, France

## Abstract

Fungi regulate key nutrient cycling processes in many forest ecosystems, but their diversity and distribution within and across ecosystems are poorly understood. Here, we examine the spatial distribution of fungi across a boreal and tropical ecosystem, focusing on ectomycorrhizal fungi. We analyzed fungal community composition across litter (organic horizons) and underlying soil horizons (0–20 cm) using 454 pyrosequencing and clone library sequencing. In both forests, we found significant clustering of fungal communities by site and soil horizons with analogous patterns detected by both sequencing technologies. Free-living saprotrophic fungi dominated the recently-shed leaf litter and ectomycorrhizal fungi dominated the underlying soil horizons. This vertical pattern of fungal segregation has also been found in temperate and European boreal forests, suggesting that these results apply broadly to ectomycorrhizal-dominated systems, including tropical rain forests. Since ectomycorrhizal and free-living saprotrophic fungi have different influences on soil carbon and nitrogen dynamics, information on the spatial distribution of these functional groups will improve our understanding of forest nutrient cycling.

## Introduction

Ectomycorrhizal (ECM) and saprotrophic fungi are major contributors to nutrient cycling in forest ecosystems [1]. These functional groups are globally distributed and coexist in many forest ecosystems. Approximately 6000 tree species worldwide depend on ECM fungi for nutrient acquisition [2], and the distribution of ECM trees spans the globe ranging from northern boreal regions to tropical rain forests. Strikingly, a disproportionate number of the dominant trees in temperate, boreal and certain tropical forests form ECM associations [3]–[6], suggesting that ECM fungi are likely responsible for a significant quantity of C, N, and P cycling worldwide. In boreal forests, ECM fungi contribute up to 86% of total plant N [7]. Saprotrophic fungi are also critical to nutrient cycling, and are the major decomposers of complex, organic molecules such as lignin. Thus, understanding how ECM and saprotrophic fungi are distributed within and across ecosystems is critical for making inferences about nutrient cycling and related ecosystem functions in forest communities.

It is well established that mycorrhizal fungi interact with other soil organisms such as bacteria and invertebrates, but interactions among mycorrhizal and decomposer fungi have been more challenging to evaluate [1], [8]. There is evidence from boreal and temperate forests that ECM and saprotrophic fungal taxa vertically segregate in soils [9]–[11], suggesting physiological specialization of fungi on organic substrates in various levels of decay [10]. However, there have been few studies of fungal spatial dynamics in tropical ECM forests, so it is unclear if the patterns detected in boreal and temperate forests are similar to those found in the tropics.

While the majority of trees in temperate and boreal forests form ECM associations, most species of trees in lowland tropical rain forests form arbuscular mycorrhizal (AM) associations. When tropical trees do form ECM symbioses, they are more likely to become locally dominant [6] or in some cases regionally dominant (e.g., the Dipterocarpaceae in Southeast Asia). At this point, we do not know if generalizations can be made about ECM forests at a global scale or if tropical ECM forests contain unique fungal communities that function differently from ECM fungi at higher latitudes. From the data that have been collected, it seems that tropical forests have lower ECM diversity than temperate and boreal ecosystems [12], [13], although there is clearly a gap in our knowledge and a paucity of belowground studies in tropical ECM forests. Since tropical forests harbor 40% of all terrestrial biomass and are responsible for 32% of terrestrial net primary production [14], [15], understanding the dynamics of fungal distribution and function in tropical forests is important for making inferences about global nutrient cycles.

In this study, we used sequence-based approaches to assess the distribution of fungal taxa in a tropical forest located in central Guyana and a boreal forest in Delta Junction, Alaska. The tropical forest site contained two types of rain forest: an ectomycorrhizal monodominant forest and a non-ectomycorrhizal mixed forest [16], [17]. Our objectives were to: 1) examine the level of taxonomic similarity in fungal community composition across the two ECM forests in different biomes, 2) compare fungal community composition across organic and mineral soil horizons within each ecosystem, and 3) determine if patterns of functional group separation across soil horizons were analogous in the boreal and tropical forest. Since tropical ECM forest dynamics have been shown to be significantly different than non-EM forests within the same biome [6], [18], we predicted that the boreal forest and tropical ECM forest would exhibit more similar fungal community patterns than the ECM and non-EM tropical forest.

## Materials and Methods

### Study Site and Sample Collection

Samples used for this study were collected from sites in Alaska, USA (63°55′N, 145°44′W) and Guyana (5°4′ N, 59°58 W) between 2007 and 2009. In Alaska, the site consisted of boreal spruce forest that has not burned in over 80 years [19].The forest canopy was dominated by *Picea mariana* (Mill.) Britton, Sterns & Poggenb. (Pinaceae), which forms ECM associations and comprises the vast majority of the canopy trees [19], [20]. Likewise, the ECM forest site in Guyana consisted of mature forest dominated by the ECM tree *Dicymbe corymbosa* Spruce ex. Benth (Caesalpiniaceae), in which *D. corymbosa* comprises up to 90% of canopy trees [16], [21]. *Dicymbe corymbosa* was also the only ECM host in the plots used for this study, thereby making it an ideal comparative site to the *Picea*-dominated boreal forest. As a non-ECM comparison, three plots from mixed forest in Guyana were also analyzed, which do not contain dominant ECM species [17]. Since the majority of boreal forest trees are ECM, we did not have a non-ECM forest comparison for the boreal biome. Permits for the field research in Guyana were granted by the Guyana Environmental Protection Agency and the Ministry of Amerindian Affairs. The sites were not located on private or protected land and did not involve endangered or protected species.

In each ecosystem, samples were separately collected from the litter and upper soil horizons (0–20 cm) from previously established plots at both sites. Plots in both sites were at least 100 m apart. In the boreal forest, a total of three plots were sampled, with each plot having dimensions of 10×10 m. One composite soil sample was derived from five soil cores taken from each plot. These plots were also used as control sites in a previous study [22]. In the tropical forest, ten composite soil samples were taken from three previously established forest plots (30×100m) in the *Dicymbe*-dominated forest [21]. At the same points of soil core sampling, we also collected litter samples from the forest floor. Plot sizes across sites were different, as these study sites were established independently without the original intention of comparative analyses. However, since samples were collected in a similar manner and DNA was extracted with the same protocol, the extracts were sequenced together for comparative analyses of vertical fungal separation, rather than comparisons of fungal species richness.

To evaluate litter fungi in a more controlled way so that only the dominant tree litter was used, we set out freshly fallen leaf litter in mesh bags on the forest floor at both sites. In the boreal forest, 4 g air-dried *Picea mariana* leaf litter was placed in litter bags composed of 2 mm mesh (window screen) lined with 0.5 mm mesh (bridal veil) to prevent loss of needle fragments. Leaf litter in the mesh bags from Guyana was composed of 10 g air-dried, freshly-fallen *D. corymbosa* leaves. After one year of incubation on the surface of the forest floor, decomposed litter from six bags in the boreal forest and ten bags in the tropical forest was transported to the laboratory, where it was frozen at –80°C until analysis. All samples remained frozen during transport, which was less than 10 h for both sites.

### Molecular Analyses

To examine fungal community composition across ecosystems, we first analyzed environmental soil and litter samples from the six plots at each site using 454 pyrosequencing. To homogenize the soil samples, each composite sample was passed through a 2 mm sieve that had been sterilized with ethanol and 15 min of uv radiation. All homogenizations were accomplished in a sterile, benchtop PCR hood (AirClean Systems, Inc, Raleigh, NC). Litter was hand homogenized with sterile gloves. Since the litter was highly decomposed, mechanical grinding was not necessary. From each composite soil and litter sample for each plot, total DNA was extracted from three 0.25g subsamples to obtain a representative sample [23] using a Powersoil DNA extraction kit (MoBio, Carlsbad, CA) according to the manufacturer’s instructions. These three DNA extracts were pooled to create one representative soil DNA extract. General fungal primers (SSU817f and SSU1196r) targeting a portion of the 18S rRNA gene were modified for 454 sequencing [24]. PCR amplifications were done as described previously [24]–[26] with 30 mM of each primer (0.25 µl), 22.5 µl Platinum PCR SuperMix (Invitrogen, Carlsbad, CA), and 3 µl of DNA template. Three PCR reactions per sample were pooled for analysis. PCR products were sequenced at the Environmental Genomics Core Facility at the University of South Carolina (Columbia, SC) on a Roche 454 Gene Sequencer with Titanium chemistry. Sequenced amplicons were quality checked, aligned, and grouped into operational taxonomic units (OTUs) at a 97% sequence similarity cutoff with the Quantitative Insights Into Microbial Ecology (QIIME) pipeline [27]. The centroid sequence from each OTU cluster was chosen and used to create a phylogenetic tree with the FastTree algorithm [28]. Taxonomic information for each OTU was determined using the BLAST algorithm [29] against identified sequences in both Genbank and the SILVA database [30]. Ultimately, we used an open-reference for OTU picking and sequences <400 bp were removed. The (phred) quality score cutoff was 25 and sequences containing ambiguous characters and those having an unreadable barcode were also removed. Non-fungal sequences were manually removed following taxonomic assignment. The average sequence length was ∼450 bp. Fungal sequences have been deposited in the Sequence Read Archive of Genbank (Accession # SRP009079.1).

To gain more detailed taxonomic information about soil and litter fungi, we used clone library sequencing on the same soil samples analyzed in the pyrosequencing runs and on litter from incubated leaf litter bags to standardize for litter species and decomposition time. The same DNA extracts used in pyrosequencing were analyzed for the composite soil samples and DNA from litter bags was extracted with a PowerSoil DNA kit (Mo Bio Laboratories, Inc, CA) as described above. Three DNA extractions of each sample were again pooled for each site. Fungal DNA was selectively amplified from soil and litter DNA extractions using the ITS1-F forward primer [31] and the TW13 reverse primer [32]. These primers target ∼600 bp of the ITS region and ∼700bp of the 5′ portion of the 28S region. The reason for choosing these primers is that amplification of the 28S region allowed for alignment of amplicons and phylogenetic community analysis, whereas the hypervariable ITS region allowed for higher taxonomic resolution at the subgeneric level [31], [33]. PCR reactions were carried out in 30 µL volumes with 200 mM Tris-HCl PCR buffer, 1.23 mM MgSO_4_, 0.2 mM each dNTP, 0.5 µg µL^−1^ BSA, 0.1 µM each primer, and 0.01 U µL^−1^ Platinum *Taq* DNA Polymerase (Invitrogen, Carlsbad, CA), and 0.13 µL template DNA uL^−1^ reaction volume. PCR reactions were done in an iCycler thermocycler (BioRad) with the following program: 5 min initial denaturation at 95°C, followed by 35 cycles of 30 sec at 95°C, 45 sec of annealing at 50°C, 6 min of elongation at 72°C, and a final elongation for 10 min at 72°C. While these PCR conditions are frequently used in the literature, we acknowledge that the high cycle number may have skewed the mixed template amplifications in favor of more abundant groups [34], [35].

PCR products were gel-purified by running each sample on a 1.5% agarose gel; target bands were cut from the gel and cleaned up with a Qiaquick gel extraction kit (Qiagen, Valencia, CA). Clone libraries were constructed with the gel-purified PCR products using the Topo TA Cloning Kit for Sequencing with PCR 4-TOPO vector (Invitrogen) following the manufacturer’s instructions. This vector allowed for blue/white colony screening, such that only chemically competent E. coli cells that were white in color were selected for sequencing. We picked 96 colonies from each clone library, 384 sequences total for a total of 4 clone libraries; one for each organic soil fraction in each ecosystem to identify the dominant fungal taxa. Clones were bi-directionally sequenced at the Laboratory for Genomics and Bioinformatics at the University of Georgia (Athens, GA).

Raw DNA sequences were edited using using CodonCode Aligner version 2.0 (CodonCode Corporation, Dedham, MA) and Bioedit. Contiguous sequences were constructed for forward and reverse DNA sequences using Geneious version 3.7.0 (Biomatters Ltd., Auckland, New Zealand). Contiguous sequences have been deposited to Genbank (Accession #: JN889716 - JN890544). Alignments were made in ClustalW [36] using only the 28S portion of the DNA, as the ITS portions are too variable for alignment. Distance matrices were generated using the default parameters of Phylip DNADIST [37]. 28S sequences having ≥ 99% sequence similarity, as determined by DOTUR [38], were assigned the same Operational Taxonomic Unit (OTU). OTUs were assigned to taxa using the BLASTn algorithm against known sequences in GenBank [29] and the UNITE database [39]. For all BLAST searches the full consensus sequences were used, rather than just the 28S portion, for better identification resolution. A taxonomic name was assigned to an OTU only if the name occurred within the top ten best BLAST matches, query coverage was >95%, and the e-value was 0.0. If the top ten matches were all ‘uncultured’ or ‘unidentified’, then ‘unknown’ was assigned to the OTU. Chimeras were identified by separately BLAST searching the 28S and ITS regions; if the top five hits in GenBank did not match for both regions of DNA, the sequence was considered chimeric and discarded. Functional group assignments (saprobe, EM, pathogen, etc.) were given to OTUs with assigned identities only if the taxonomic affiliation could reliably be placed in a group where the majority of species are known to have that particular function. For a few groups (notably Amanitaceae, Entolomataceae, and Clavulinaceae), the ECM function was assigned since it is the dominant function of that family or if the OTUs aligned to genera known to be ECM, even though there are some cases of fungal taxa in those families that can be saprotrophic [40], [41]. Ambiguous genera or families in which there was not a predominant function were listed as unknown functional groups.

### Statistical Analyses

To determine differences in fungal community composition across soil and litter horizons in the forest plots, fungal sequences were rarified to 1000 sequences [42] and proportional counts of sequences per OTU group were then square-root transformed to minimize the influence of rare taxa. OTU abundance data were then analyzed by generating distance matrices with the Bray-Curtis coefficient followed by Analysis of Similarity [ANOSIM; 43] using Primer-version 6 software (Primer-E, Plymouth, UK). Nonmetric multidimensional scaling plots and dendrograms were used to visualize similarity in fungal community composition across sites and horizons. In the ECM forests at both sites, the relative proportions of the most abundant ECM fungal families were analyzed across sites and horizons (litter versus soil) using a multivariate general linear model. For the clone library data, a two-way ANOVA was used to assess differences in fungal taxonomic richness between soil horizons and recently-shed litter within and across ecosystems.

## Results

We obtained 31,942 sequences from pyrosequencing with an average of 1330 sequences per sample and approximately 450 bp in length. Prior to downstream analyses, all non-fungal and unclassifiable sequences were removed, which represented approximately 7% of the sequences. Thus, a total of 29,837 sequences were used for downstream analyses. Of the sequences that could be identified as fungi from the 18S pyrosequencing data, an average of 327 unique operational taxonomic units (OTUs) were observed for each sample. Across all samples, 28% of sequences were Ascomycota, 55% were Basidiomycota, 9% were Chytridiomycota, 1% were Glomeromycota, 1% were basal fungal lineages, and 5% could not be assigned to a phylum. The inability to assign a phylum to these sequences may in part be due to the presence of deeply diverging fungal lineages that have not yet been characterized in Genbank [44].

**Figure 1:**
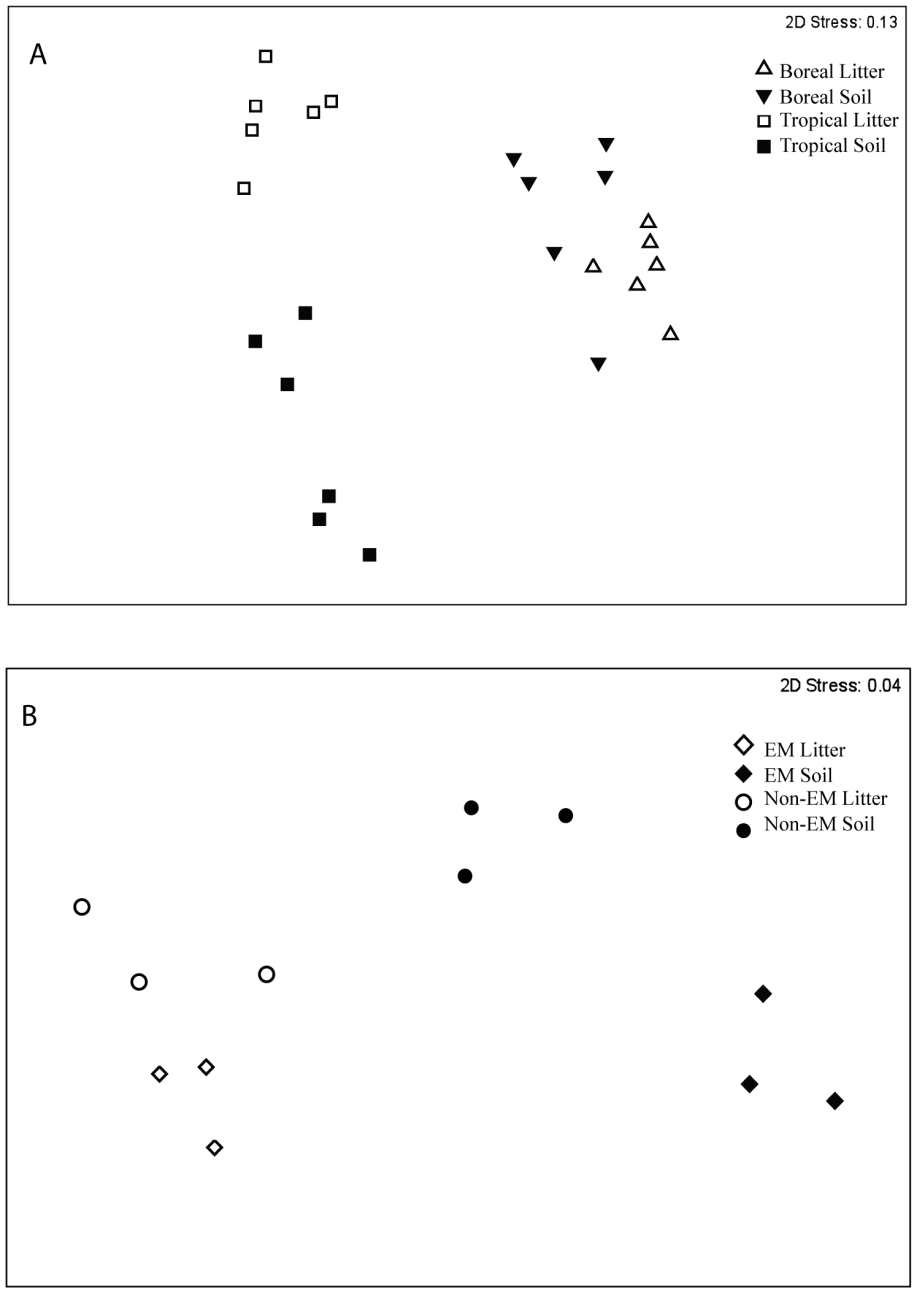
Non-metric multidimensional scaling plot for pyrosequencing OTUs in litter and soil horizons based on Bray-Curtis distance across sites (A) and across forest types (EM vs. non-EM) in the tropical ecosystem (B).

Ordination of pyrosequencing data showed that fungal communities were distinct across tropical and boreal ecosystems and across horizons within site (Fig. 1A). These patterns were confirmed by ANOSIM for both site (*P* = 0.02) and horizon (litter versus soil) within site (Alaska *P*<0.01; Guyana *P*<0.001). When fungal communities in the tropical forests were analyzed separately from boreal samples, fungal taxa in the ECM forest were distinct from the non-EM forest across horizons in each forest type (*P*<0.001; Fig. 1B).

**Figure 2:**
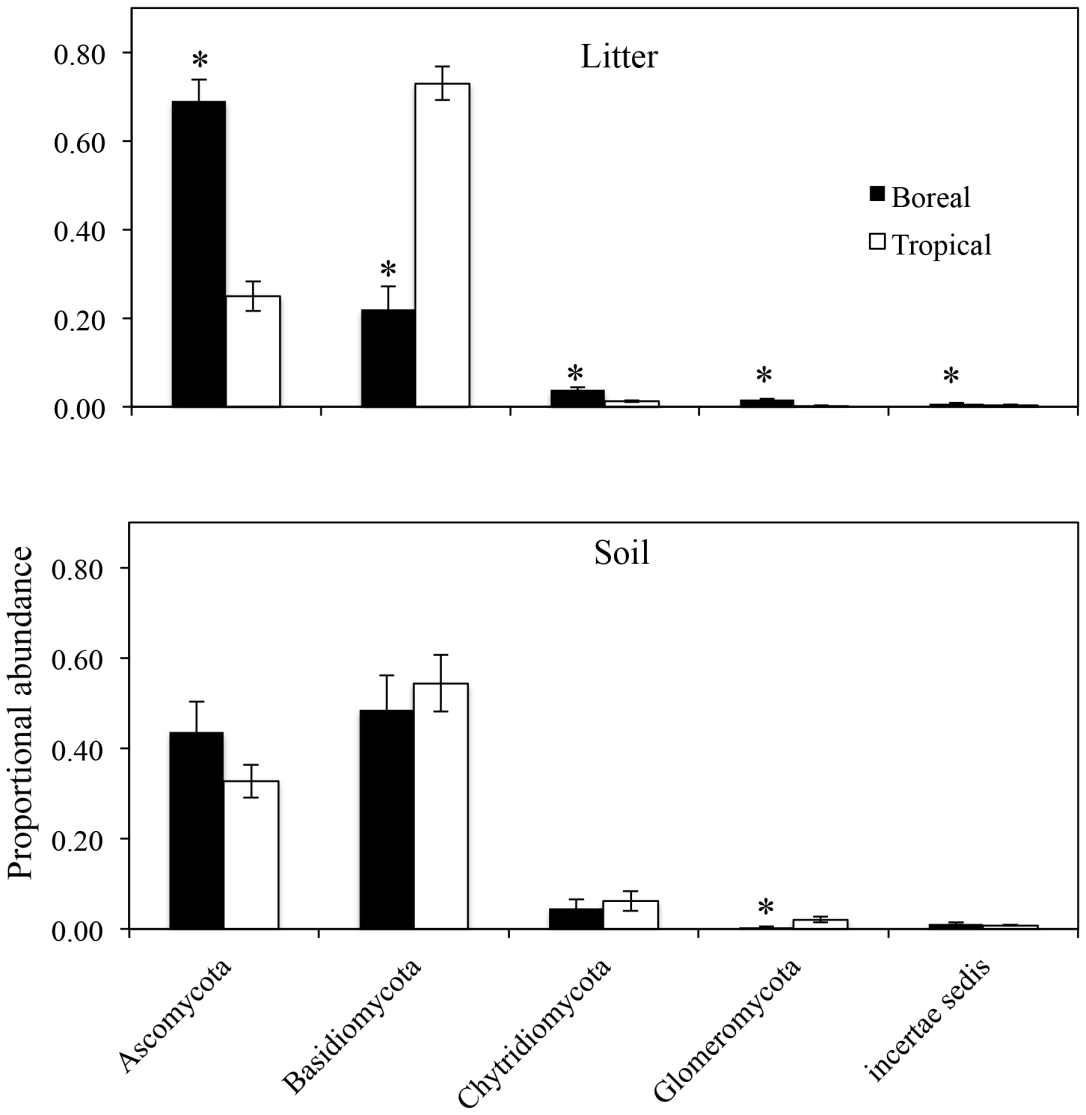
Proportional abundances of fungal taxa from pyrosequencing data assigned to each phylum across sites and horizons. Asterisks denote significance between boreal and tropical forests at *P*<0.05.

**Figure 3:**
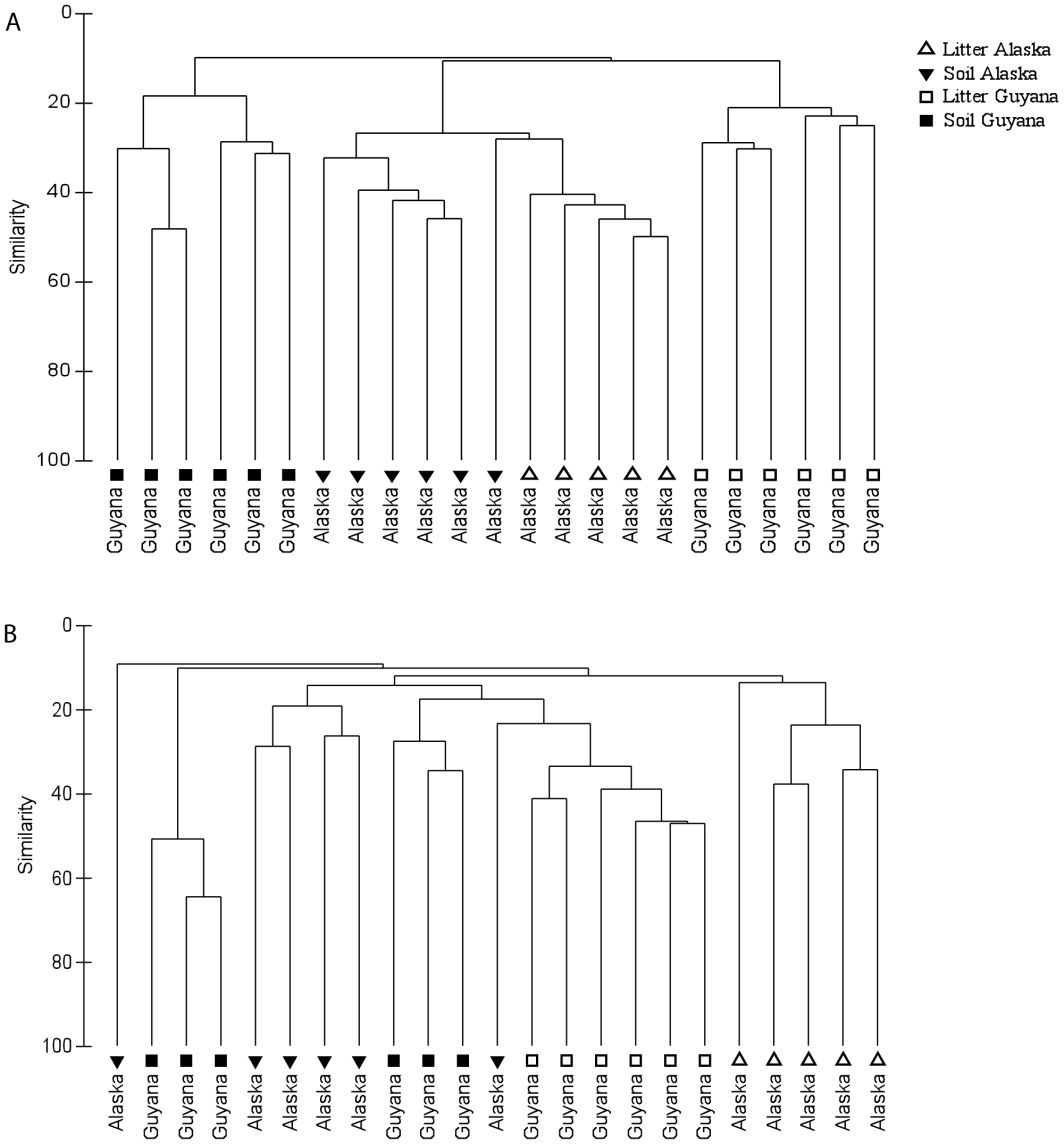
Dendrograms derived from cluster analyses are shown for pyrosequencing OTUs identified as Ascomycota (A) and Basidiomycota (B). Similar clustering patterns by site (Guyana versus Alaska) and horizon was observed for fungal communities in both phyla.

When pyrosequencing-derived fungal OTUs from soil and litter samples were compared across tropical and boreal sites, the proportional abundances of fungal phyla were significantly different in litter samples (*P<*0.01 for all comparisons; Fig. 2). However, the proportional abundance of fungal phyla in soil samples were not significantly different, with the exception of the Glomeromycota (F (1,11) = 7.4, *P* = 0.02), which was more abundant in the tropical soils. The Ascomycota and Basidiomycota comprised 80–90% of fungal OTUs in both horizons at both sites. Thus, to determine if these phyla were differentially driving the observed biogeographical patterns of fungi, we separately analyzed OTUs assigned to each phylum. Cluster analysis showed that fungi in both phyla displayed similar patterns across sites and horizons (Fig. 3).

**Figure 4:**
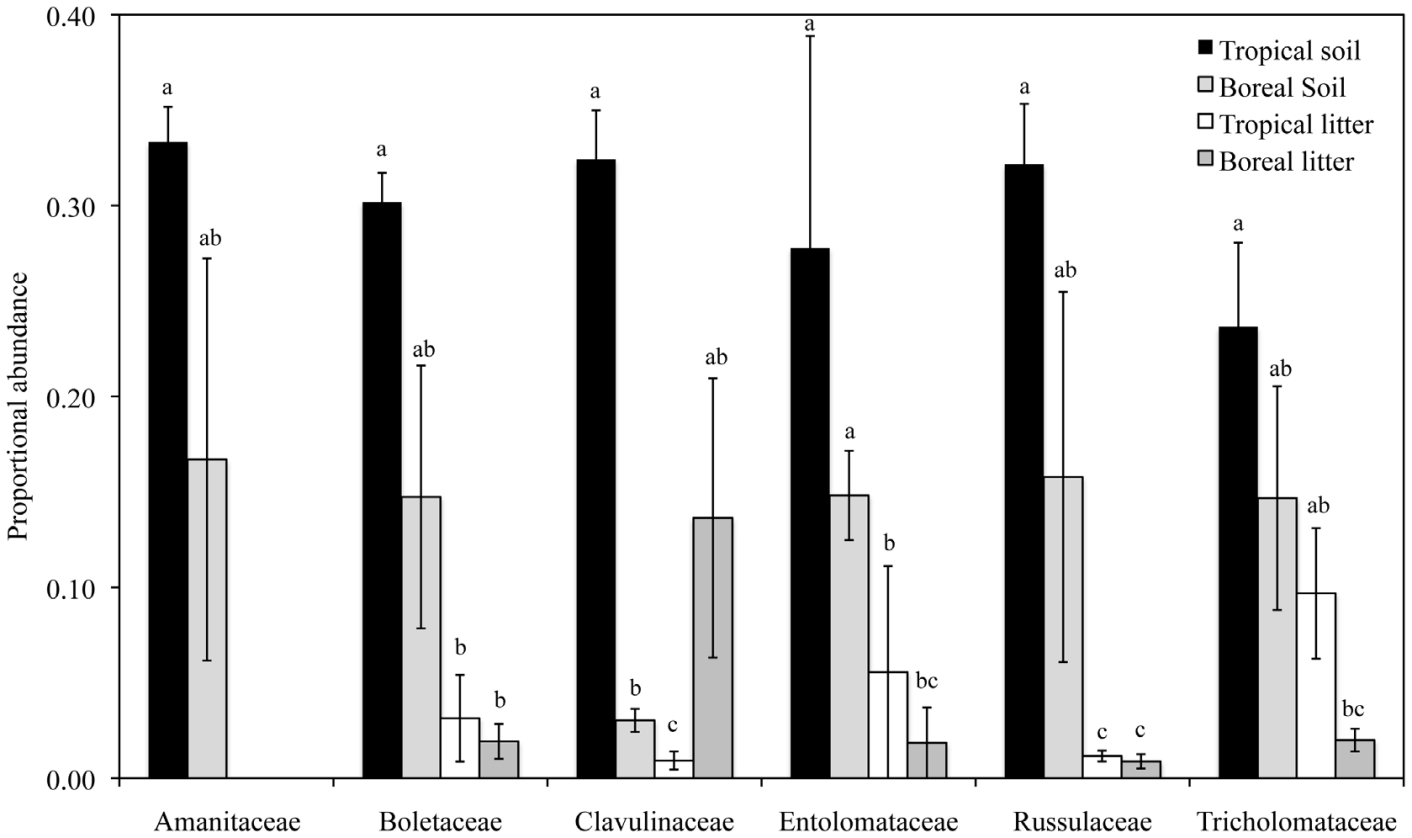
Proportional abundances of sequences derived from 454 pyrosequencing (calculated on a per-sample basis using total sequences as the denominator) of predominantly ectomycorrhizal fungal families found to be abundant in the boreal and tropical forests. Different letters indicate significance levels at *P*<0.05.

While pyrosequencing data provide limited taxonomic resolution as the sequences cannot be reliably identified beyond the family level, some fungal families are exclusively or mostly ECM and could be compared across ECM forests. In both boreal and tropical ECM sites, there were six predominantly ECM fungal families that were among the 10 most abundant ECM families in both litter and soil horizons collected from the plots (in terms of sequence abundance), so these taxa were used to compare ECM communities across the boreal and tropical ECM forests (the non-EM tropical forest was excluded, as very few ECM taxa were detected). The relative abundance of these six predominantly ECM families was not significantly different across boreal and tropical forests with the exception of the Clavulinaceae, which had higher abundance in the tropical ECM forest (F (1,8) = 237.0; *P*<0.001; Fig. 4). Of these six ECM families, there were significantly more ECM taxa detected in soil compared to litter for all families except for the Clavulinaceae, which had a high relative abundance in the boreal litter.

Clone library sequencing generated a total of 119 unique OTUs out of 329 analyzed sequences; 56 of these OTUs were detected from the boreal forest and 63 were detected from the tropical rain forest. From the original 384 sequences, a total of 55 sequences were discarded based on poor sequencing quality and chimera formation (5 chimeras detected). In both ecosystems there were more Basidiomycota than Ascomycota in the O_a_ (top mineral) horizons, but approximately equal representation of Ascomycota and Basidiomycota in the recently shed leaf litter. We found a total of 44 Ascomycota (22 in the boreal forest, 22 in the tropical forest), 73 Basidiomycota (34 in the boreal forest, 39 in the tropical forest) and 2 unknown taxa, both detected in the tropical forest.

Based on alignments unknown clone library sequences in GenBank using the BLASTn algorithm [29], the dominant fungal taxa were found to be distinct in each organic soil horizon. In the boreal forest, the fungal community from the upper soil horizons (0–20 cm) was dominated by the Agaricales, and particularly, the ECM genus *Cortinarius*. The next most abundant identified order in the boreal soil was the Helotiales, from sequences closely matched to pathogenic fungi. This order was also the second most abundant order of fungi in the boreal litter bag samples. The most abundant order on the boreal litter was the Microbotryomycetes (incertae sedis), specifically *Zymoxenogloea* sp., of which little ecological information is known. The Agaricales were the most common order of fungi found in both the soil and litter bag samples in the tropical rain forest.

**Figure 5:**
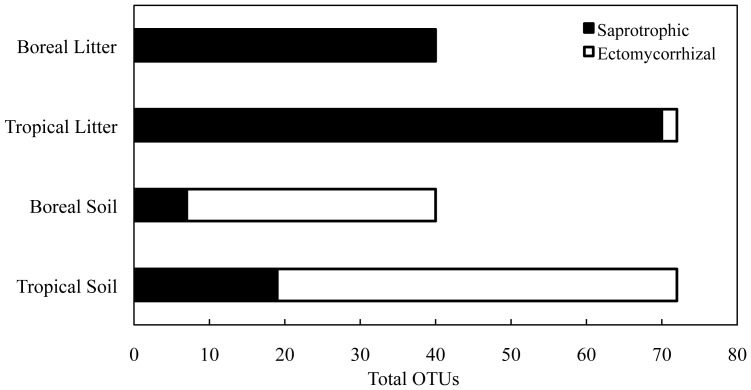
Distribution of clone library OTUs aligning with saprotrophic (black bars) and ectomycorrhizal fungi (white bars) across organic litter and underlying soil horizons (0–20 cm) in the boreal and tropical forest ecosystems. OTUs for this analysis were derived from clone library sequencing.

Since ecological function could only be assigned to exclusively ECM families in the pyrosequencing data, we used the information from the clone library sequence identifications to determine how the dominant ECM and non-EM fungi were distributed. We were able to assign an ecological function (EM fungus, saprotroph, pathogen, etc.) to 96 of the 119 unique OTUs from clone library sequencing, as inferred from the GenBank sequence alignments. We found that saprotrophs occurred in recently-shed leaf litter and ECM fungi in underlying soil horizons (0–20 cm) in both the boreal and tropical ecosystem (Fig. 5).

## Discussion

While numerous sporocarp surveys have been done in tropical forests [e.g., 45,46,47], our study provides some of the first molecular evidence that confirms biogeographical separation of fungal communities across a tropical and boreal forest, despite the occurrence of dominant trees that form ectomycorrhizae in both ecosystems. As has been found in other tropical ECM forests, the major ECM fungal lineages reflect those already known to dominate temperate and boreal ecosystems [48], [49]. Additionally, fungal communities were unique across soil and litter horizons within the same ecosystem, possibly due to fungal specialization on substrates in differing levels of decay [50]. Clone library sequencing and pyrosequencing showed analogous results in both ecosystems indicating that these patterns are robust to sequencing technology and gene region targeted, which has been a major concern among microbial ecologists [51]. While the clone library sequencing gave more reliable taxonomic information for the environmental DNA sequences (i.e., longer sequence reads), the OTUs generated from pyrosequencing aligned to similar taxa, probably as a result of incomplete coverage of fungal reference sequences in Genbank. However, because pyrosequencing allows for greater sequencing depth (for this study samples were rarified to 1000 sequences each), we can more reliably say that we have fully characterized the fungal community of a sample. Thus, the tandem use of these technologies provides strong support for our results in terms of fungal community characterization and taxonomic placement of environmental sequences. Another result supported by both pyrosequencing and clone library sequencing was that within the tropical and boreal ECM forests, ECM fungi were not prevalent in litter horizons from the forest floor, but rather occupied lower organic and mineral soil horizons.

Findings that ECM fungi were more abundant in deeper soil depths have also been observed in temperate [9] and Swedish boreal forests [11], indicating that vertical segregation of ECM and saprotrophic fungi in soils may be a widespread phenomenon in ECM-dominated forest ecosystems. The reasons for spatial segregation of these fungal groups are likely due to the distribution of C and nutrients in litter versus soil. Since ECM fungi have decomposer abilities [52], [53], but are not C-limited like other saprotrophs due to their access to plant photosynthate, ECM fungi may reside below the freshly-fallen litter layer in deeper horizons to target substrates richer in other nutrients [54], [55]. An alternative, but not mutually exclusive explanation for the predominance of ECM fungi in the soil horizons may be due to antagonistic relationships between ECM and saprotrophic fungi [56]–[58]. Since these fungal groups compete for some of the same resources, they may vertically segregate to avoid competitive exclusion [10].

Within the tropical ecosystem, pyrosequencing showed that soil fungal communities were distinct between the ECM and the diverse, non-ECM forests, indicating that at a local scale, the presence of an ECM tree can dramatically alter the general fungal community. The magnitude of differentiation in soil fungal communities across these tropical forests was almost as dramatic as the differentiation observed across biomes, and previous research in this site has shown that soil physicochemical properties are not responsible for determining these patterns [18]. Fungi detected in forest floor litter were also clustered by forest type in the tropical system, although the magnitude of difference was much less. This result may be due to the fact that the same species of non-ECM trees are present in both tropical forests [16], [21], so the chemical composition of the leaf litter is somewhat similar [18]. However, there is an overwhelming abundance of litter in the ECM forest from the ECM, monodominant tree (*Dicymbe corymbosa*), which may explain the differences in the litter fungal communities across these forests. In another study, a reciprocal litter decomposition experiment has shown that leaf litter of *Dicymbe* and non-ECM trees decomposes slower in the ECM forest relative to the non-ECM forest [18], indicating that these differences in fungal communities may result in altered nutrient cycling.

In the boreal biome, the results of this study suggest that related fungal taxa may dominate the organic layers in boreal forest soils across different systems. For example, the genus *Cortinarius* was the most abundant in our boreal soil samples, and this genus also dominates Swedish boreal forest soil [11]. In an earlier study, Allison et al. [59] also found that the ECM genus *Cortinarius* was the dominant taxon from our Alaskan study site. Future work focusing on the function of *Cortinarius* in decomposition would be valuable, as it is a globally distributed genus and known to occupy litter at late stages of decomposition [60]. However, other than protease ability [61], its complete enzymatic capabilities are still unknown. We also found *Cortinarius* taxa in the tropical samples, although our sequence analysis indicated that they are different genotypes than the boreal taxa.

Some of the ECM genera we detected from the Agaricales in the tropical samples are known to associate with the dominant ECM tree, *Dicymbe corymbosa*, as they have been described by mycologists working in that region [62], [63]. However, some of the sequences we generated are likely undescribed taxa. This is probably true for the numerous *Clavulina* species we observed from the Cantharellales in the tropical soil, which was the second most abundant order. *Clavulina* diversity is known to be high in this region [64], which reflects what we detected in our environmental pyrosequencing data.

The finding that fungal communities are distinct in litter horizons also has implications for environmental sampling of fungal communities. For a comprehensive understanding of microbial community composition, sampling should incorporate both the organic and underlying soil horizons. In addition, environmental changes that affect one soil layer more than another may have disproportionate consequences for the two fungal groups. For instance, forest fires primarily burn the upper soil horizons (depending on severity), so direct effects of fire may be stronger on saprotrophic fungi than on ECM fungi. Making inferences about fungal communities from only mineral samples may, therefore, underestimate diversity and provide an incomplete picture of community composition.
